# Supplementary material for: A UMLS-based spell checker for natural language processing in vaccine safety
Source: BMC Med Inform Decis Mak. 2007 Feb 12;7:3. doi: 10.1186/1472-6947-7-3 (PMC1805499; doi:10.1186/1472-6947-7-3)
Supplement: Additional file 13 — RAP application directory. Sets up directory for RAP (RDF application for PHP) source code [file 1472-6947-7-3-S13.gz › rap/doc/phpdoc/classtrees_syntax.html]

xml version="1.0" encoding="iso-8859-1"?


## Root class GRDDLParser

- Object **(Different package)**
  - GRDDLParser

## Root class N3Parser

- Object **(Different package)**
  - N3Parser

## Root class N3Serializer

- Object **(Different package)**
  - N3Serializer

## Root class NTripleSerializer

- Object **(Different package)**
  - NTripleSerializer

## Root class RdfParser

- Object **(Different package)**
  - RdfParser

## Root class RdfSerializer

- Object **(Different package)**
  - RdfSerializer

Documentation generated on Thu, 7 Jul 2005 13:41:08 +0200 by phpDocumentor 1.3.0RC3
